# Protein N-glycosylation aberrations and glycoproteomic network alterations in osteoarthritis and osteoarthritis with type 2 diabetes

**DOI:** 10.1038/s41598-022-10996-1

**Published:** 2022-04-28

**Authors:** Yi Luo, Ziguang Wu, Song Chen, Huanhuan Luo, Xiaoying Mo, Yao Wang, Jianbang Tang

**Affiliations:** 1 School of Basic Medicine, Guangzhou University of Chinese Medicine, Guangzhou, 510006 Guangdong Province China; 2 Zhongshan Hospital of Chinese Medicine, No. 3 Kangxin Road, Xi District, Zhongshan, 528400 Guangdong Province China; 3 Science and Technology Innovation Center, Guangzhou University of Chinese Medicine, Guangzhou, 510405 Guangdong Province China

**Keywords:** Diseases, Medical research

## Abstract

Whether the relationship between type 2 diabetes mellitus (T2DM) and osteoarthritis (OA) can be solely attributed to the shared risk factors, such as obesity, remains controversial. Several studies have revealed the critical role of abnormal glycosylation in the pathogenesis of OA and T2DM. Therefore, we speculate that T2DM may contribute to the pathogenesis of OA through the intrinsic mechanisms of N-glycosylation aberrations. Using N-glycoproteomics, we compared the changes in N-glycosylated protein abundance in cartilage samples from patients with OA without and with T2DM (DM-OA), and from patients with traumatic joint injury (NC) as controls. We identified 847 N-glycosylation sites corresponding to 729 peptides fragments from 374 proteins. The number of N-glycosylated proteins in the DM-OA group tended to decrease compared with that in the OA and NC groups. We identified 22 upregulated and 1 down-regulated N-glycosylated peptides in the OA group compared to the NC group, while only fibronectin 1 (FN1) at position N1007, cartilage intermediate layer protein 1 (CILP) at N346, and collagen type VI alpha 1 chain (COL6A1) at N804, were also identified in the DM-OA group. Compared to the OA group, the downregulation of secreted protein acidic and rich in cysteine (SPARC) at N116, collagen type VI alpha 1 chain (COL6A2) at N785, and asporin (ASPN) at N282, and the upregulation of complement component C8 alpha chain (C8α) at N437, were the most remarkable alterations in the DM-OA group. The differentially expressed N-glycosylated proteins between the OA and DM-OA groups were mainly located extracellularly and enriched in the KEGG pathways involving PI3K/Akt signaling, focal adhesion, and ECM-receptor interaction. Their predicted protein–protein interactions were also depicted. We were thus able to show the general characteristics of N-glycosylation aberrations in OA and DM-OA. Moreover, the upregulated glycosylated complement C8α in the DM-OA group might augment membrane attack complex activity, thereby exacerbating cartilage destruction. Although further confirmation is required, our hypothesis proposes a possible explanation for the deduction that T2DM is an independent risk factor for OA.

## Introduction

Osteoarthritis (OA), an age-related disease of the synovial joints, is one of the most expensive and disabling forms of arthritis^1^. OA is characterized by progressive deterioration of the articular cartilage and structural changes in the entire synovial joint^1^. Although OA has historically been viewed as a ‘wear and tear’ disease, it is now generally accepted as a low-grade inflammatory disease caused by metabolic disorders^2^. Preclinical research in animal models and clinical studies in patients with OA have shown that age, obesity, and metabolic syndrome are major risk factors for OA. However, the relationship between OA and diabetes mellitus (DM) remains unclear. There have been many reports describing the higher risk of OA in the DM than in the non-DM population^3,4^. T2DM is a risk factor for the development of severe OA, independent of age and body mass index (BMI). However, the role of T2DM remains controversial. Among the 12 studies reporting an odds ratio (OR) of the association between DM and OA, only 7 identified DM as an independent risk factor whereas the other 5 showed no association after adjusting for body mass index^3^. Therefore, shared common risk factors, including obesity^5,6^ and aging^7,8^, might explain the high co-occurrence of T2DM and OA, and whether T2DM promotes the occurrence and development of OA through an intrinsic mechanism remains unclear.

Glycosylation modification affects protein folding, stability, and biological functions, and further plays an important role in physiological function and cartilage pathogenesis. Abnormal glycosylation plays a critical role in OA pathogenesis. Changes in N-glycans of the high-mannose type were observed in both human OA and degenerative mouse cartilage^9^. OA has also been correlated with changes in the glycosylation pattern of total serum IgG^10^. Moreover, angiogenesis^11^, Notch-related inflammatory signaling pathway^12^, and other physiological and pathological processes involved in the pathogenesis of OA are also modulated by glycosylation modification. Conversely, diabetic patients display obvious abnormalities in protein glycosylation, including chitosidase (chit1)^13^ and immunoglobulin G (IgG)^14^. N-glycosylation map has been used as a biomarker to monitor the progression of early T2DM^15^. Therefore, we speculate that T2DM contributes to the occurrence and development of OA through alterations in glycosylation. N-glycosylation, a major type of protein glycosylation comprising the covalent attachment of glycans to asparagine residues, is one of the most important, chemically complex, and ubiquitous post-translational modifications in all eukaryotes^16,17^. N-glycosylation regulates a variety of cellular processes, affecting protein conformation, activity, trafficking, stability, and interactions with other cellular substances. In this study, we compared the N-glycosylation protein spectrum profiles of articular cartilage between OA patients with and without T2DM, using N-glycosylation proteomics techniques. This exploratory study provides suggestive ideas and evidence for understanding the association between T2DM and OA.

## Results

### General characteristics of the participants

A total of 10 patients with pathologically confirmed OA, including 5 with OA complicated with T2DM (DM-OA), 5 age-matched OA-only patients, and five patients with traumatic joint injury, were enrolled in this study after providing informed consent. The clinical characteristics are presented in Table S1. The OA group and DM-OA group had similar age (median 66 years [IQR, 54–70 years] versus 63 years [IQR, 34–80 years]), sex composition (40% male and 60% female versus 20% male and 80% female), and clinical stages (2 cases in stage 3 and 3 cases in stage 4 versus all 5 cases in stage 4, *P* > 0.05), while the body weight of the OA group was slightly higher than that of the DM-OA group (median 74 kg [IQR, 62–87.5 kg] versus 54.5 kg [IQR, 51.6–67 kg], *P* < 0.05) (Table S1). The fasting blood glucose level at admission in the T2DM-OA group was higher than that in the OA group (*P* < 0.05).

### N-glycoproteome profiling analysis of human cartilage from OA, DM-OA, and control patients

**Figure 1:**
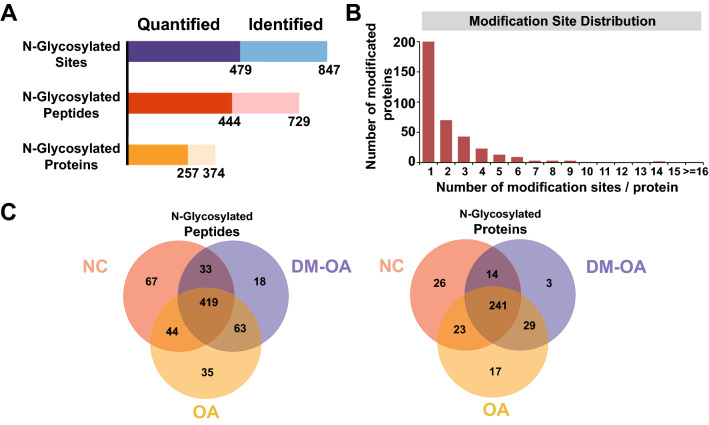
N-glycoproteome profiling analysis of human OA, OA with T2MD and control brain cartilage. (**A**) The total number of N-glycosylation sites, N-glycosylated peptides and their corresponding proteins identified in this study. (**B**) The distribution of N-glycosylation sites in N-glycosylated proteins. (**C**) Venn diagrams of identified N-glycosylated proteins and peptides indicating the number of overlapping genes in three separate pairwise comparisons.

In this project, we identified 847 N-glycosylation sites corresponding to 729 N-glycosylated peptide fragments from 374 N-glycosylated proteins. Among them, 479 N-glycosylation sites corresponding to 444 quantitative N-glycosylation peptides belonging to 257 modified proteins, were quantifiable; in at least one group, more than half of the biological repeats had the intensity value of the modified peptide. (Fig. 1A). Among the N-glycosylated proteins 46.52% possessed two or more N-glycosylation sites (Fig. 1B). Low-density lipoprotein receptor-related protein 1 (LRP1, Q07954) was the most typical protein, containing as many as 21 N-glycosylation sites (Fig. S1). The average abundance of identified N-glycosylation sites on all N-glycosylated proteins was 0.4 per 100 amino acids.

To visualize the intragroup consistency of the identified N-glycosylated peptides and proteins, Venn diagrams were constructed to indicate the number of overlapping genes in three separate pairwise comparisons. A total of 419 N-glycopeptides corresponding to 241 N-glycoproteins were shared by all three groups, whereas the NC group possessed the highest abundance of N-glycopeptides and N-glycoproteins (Fig. 1C). The numbers of distinctive N-glycopeptides identified in the NC, OA, and DM-OA groups were 67, 35, and 18, respectively, corresponding to 26, 17, and 3 distinct N-glycoproteins, respectively (Fig. 1C). Venn diagrams of individuals within each group were also drawn to show the inter-group variations in N-glycosylated peptides and proteins (Fig. S2).

### N-glycosylated proteins with differential abundances in the ALL-OA group

Using the criteria of fold change (FC) > 2 times and unadjusted *P*-value < 0.05 (Wilcoxon test), we identified 13 upregulated N-glycosylated peptides (corresponding to 10 proteins) and 2 downregulated N-glycosylated peptides in the ALL-OA group compared to the NC group (Table 1). Among the 10 enriched N-glycosylated proteins in the ALL-OA group compared to the NC group, the top three were cartilage intermediate layer protein 1 (CILP), immunoglobulin heavy constant mu (IGHM), and lumican (LUM), whereas the abundances of N-glycosylated fibrillin-1 (FBN1) and collagen type VI alpha 1 chain (COL6A1) were significantly decreased.

**Table 1:** N-glycosylated proteins with differential abundance were identified in ALL-OA (OA & DM-OA) group compared to the normal control group.

Protein	Gene	Protein name	N-glycosylated Positions	ALL OA/NC	*P* value^a^
O75339	CILP	Cartilage intermediate layer protein 1	346	10.7	0.009
P01871	IGHM	Immunoglobulin heavy constant mu	46	7.7	0.033
O75339	CILP	Cartilage intermediate layer protein 1	129	5.2	0.028
P51884	LUM	Lumican	249	4.5	0.024
P02751	FN1	Fibronectin	542	4.4	0.036
Q08431	MFGE8	Lactadherin	64	4.2	0.036
Q4LE39	ARID4B	AT-rich interactive domain-containing protein 4B	743	3.9	0.007
P01008	SERPINC1	Antithrombin-III	224	3.8	0.034
Q4LE39	ARID4B	AT-rich interactive domain-containing protein 4B	744	3.3	0.013
P05090	APOD	Apolipoprotein D	98	3.3	0.019
P10909	CLU	Clusterin	86	3.1	0.028
P02751	FN1	Fibronectin	1007	2.5	0.008
P08962	CD63	CD63 antigen	150	2.3	0.030
P35555	FBN1	Fibrillin-1	1581	0.4	0.028
P12109	COL6A1	Collagen alpha-1(VI) chain	804	0.2	0.018

^a^Wilcoxon non-parametric test.

### Alterations of N-glycosylated peptides in the cartilage of OA and DM-OA patients

**Figure 2:**
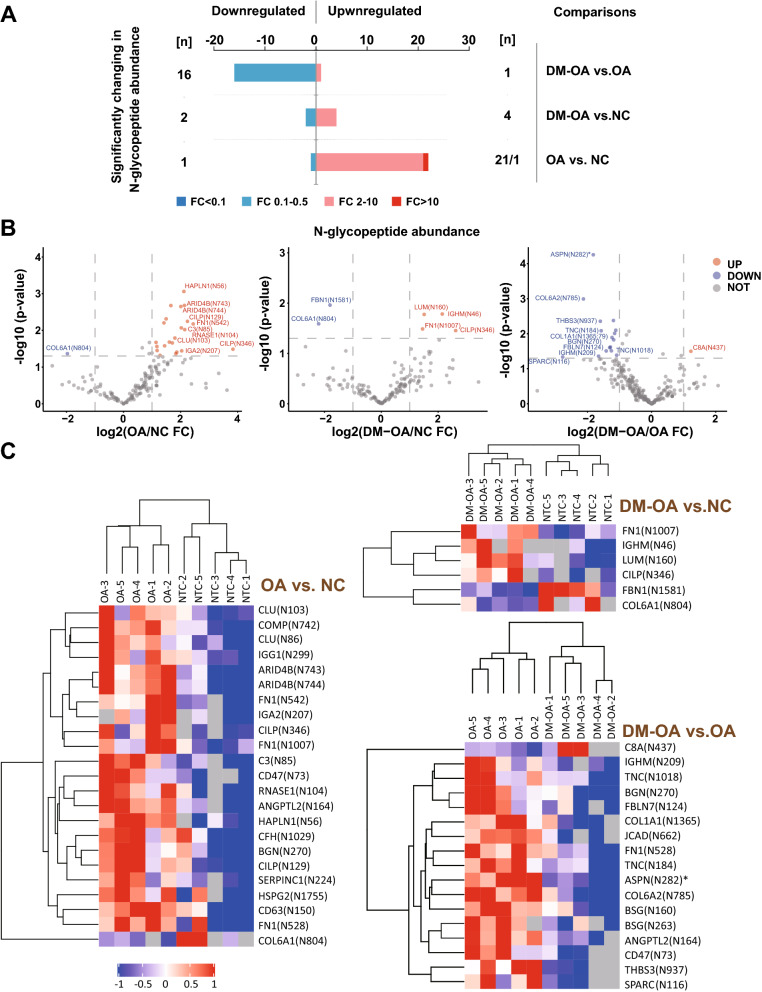
N-glycosylated peptides and their N-glycosylation sites with differential abundance were identified in pairwise comparisons of OA, DM-OA, and normal control. (**A**) The number of N-glycosylated peptides identified in pairwise comparisons of OA, DM-OA, and normal control groups with the criteria of the fold change (FC) > 2 (red) or < 0.5 (blue) and *P* < 0.05. Dark red and dark blue indicated criteria of FC > 10 or < 0.1. (**B**) Volcano diagrams for each pairwise comparison drawn by logarithmically transformed change folds versus *P*-value. (**C**) Hierarchical clustering drawn by Cluster 3.0 (http://bonsai.hgc.jp/~mdehoon/software/cluster/software.htm) and Java Treeview software (http://jtreeview.sourceforge.net) based on the N-glycopeptides abundance profiles and heatmaps (Complexheatmap R Version 3.4) showing the abundances of N-glycopeptides containing N-glycosites in pairwise comparisons, while all of these groups with the criteria of the FC > 2 (red) or < 0.5 (blue) and unadjusted *P* < 0.05. * indicate an FDR < 0.05.

Also with the criteria of fold change (FC) > 2 times and *P*-value < 0.05, we identified 22 upregulated N-glycosylated peptides (including one that increased more than tenfold) and one downregulated N-glycosylated peptide in the OA group compared to the NC group. In contrast, there were only four significantly upregulated N-glycosylated peptides, accompanied by two downregulated N-glycosylated peptides in the DM-OA group compared to the NC group. In contrast, direct comparisons between the DM-OA and OA groups showed 1 upregulated and 16 downregulated N-glycosylated peptides (Fig. 2A, Table S2). The top 10 differentially abundant N-glycosylated peptides of each pairwise comparison are shown in volcano diagrams (Fig. 2B). Unexpectedly, only the upregulation of CILP N436 and downregulation of COL6A1 N804 were shared by the OA and OA-DM groups, whereas most other upregulated N-glycosylated peptides identified in the OA group had disappeared in the DM-OA group. Using the identified N-glycosylated peptides with differential abundance, we also drew heat maps to explore intra-group and inter-group consistency. As shown in Fig. 2C, the similarities of the five samples within the same group were significantly higher than the intragroup similarity.

Interestingly, we also noted the diverse utilization of N-glycosylation sites in some proteins with multiple potential N-glycosylation sites. For example, N-glycosylation at positions 542, 528, and 1007 of fibronectin 1 (FN1) was significantly upregulated in the OA group, whereas only upregulation of FN1 with N1007 N-glycosylation remained in the DM-OA group. Similarly, the N-glycosylation of IGHM at N46 was significantly upregulated in the DM-OA group compared to the NC group, whereas its N-glycosylation at position 209/332 was significantly downregulated compared to the OA group (Table S2). We propose that the shift in N-glycosylation sites might be an important mechanism affecting the modulation of protein functions.

### Function prediction

**Figure 3:**
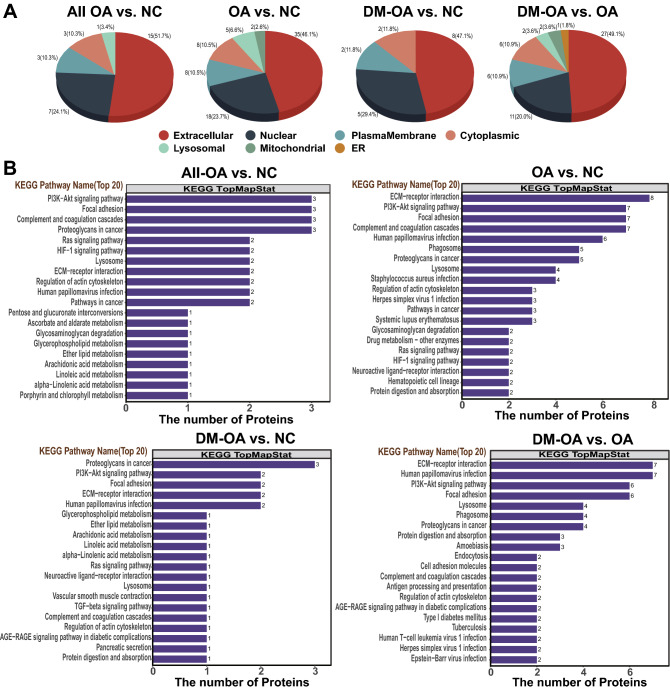
Functional annotations predicted by KEGG pathway analysis of N-glycopeptides. (**A**) Predictive subcellular organelle localization of N-glycopeptides with differential abundances identified in this study by CELLO (http://cello.life.nctu.edu.tw/). (**B**) Top 20 enriched KEGG pathways (http://geneontology.org/) of N-glycosylated proteins with different abundance identified in pairwise comparisons.

Thereafter, we tried to predict the subcellular location and function of the differential expressed N-glycosylated proteins identified in our comparisons. In order to make the analysis more reliable, we extended the criteria to include not only N-glycosylated proteins with differential abundances (with criteria described previously), but also N-glycosylated proteins presented in 3 or more samples in one group whereas absented in the other group. The statistics of N-glycosylated proteins identified in each comparison were shown in Table S3. The subcellular localization of proteins regulates their functions. Using the subcellular structure prediction software CELLO^18^ predict the subcellular location of N-glycosylated peptides with differential abundances. As shown in Fig. 3A, most of these proteins were extracellular (> 45% in all three groups) with the percentage of nuclear proteins ranging from 20 to 30%. Interestingly, proteins located in the endoplasmic reticulum were identified exclusively in the comparison between the DM-OA and OA groups.

Thereafter, the identified N-glycosylated proteins were annotated using Blast2Go (https://www.blast2go.com/)^19^ software based on the Gene Ontology (GO) database^20^. As shown in Fig. S3, the most affected N-glycosylated proteins indicated the "cellular processes" of biological process (BP), "binding" of molecular function (MC), and "extracellular region" of cellular component (CC) in all comparisons. Moreover, "metabolic process" was the second enriched BP pathway in OA group or DM-OA group, as well as in ALL-OA group, compared to NC group.

Thereafter, the Kyoto Encyclopedia of Genes and Genomes (KEGG database)^21^ was used to retrieve the involved KEGG orthology identifications and subsequently mapped to KEGG pathways. The top 20 KEGG pathways for the identified N-glycosylated proteins are shown in Fig. 3B. Compared to the NC group, the top three enriched pathways in ALL-OA group were "PI3K-Akt signaling pathway", "focal adhesion", and "Complement and coagulation cascades", while those in OA group were "ECM-receptor interaction", "PI3K-Akt signaling pathway", and "focal adhesion", and in DM-OA group were "proteoglycans in cancer", "PI3K-Akt signaling pathway", and "focal adhesion." We also noticed that those KEGG pathways shared some N-glycosylated proteins, like FN1, COL6A1, and platelet-derived growth factor c (PDGFC) which were shared by PI3K-Akt and focal adhesion pathways, as shown in Table S4. Fisher's exact test was also performed to analyze KEGG pathway enrichment. As shown in Fig. S4, compared to the NC group, "Ras" in the ALL-OA group, "drug metabolism" and "glycosaminoglycan degradation" in the OA group, as well as "proteoglycans in cancer", "vascular smooth muscle contraction" and "metabolism of alpha-Linoleic acid, Linoleic acid, ether lipid, and glycerophospholipid" in the DM-OA group, were significantly enriched. The proteins composed of the enriched pathways were annotated in Fig. S4.

### Protein–protein interaction (PPI) analysis of N-glycoprotein

**Figure 4:**
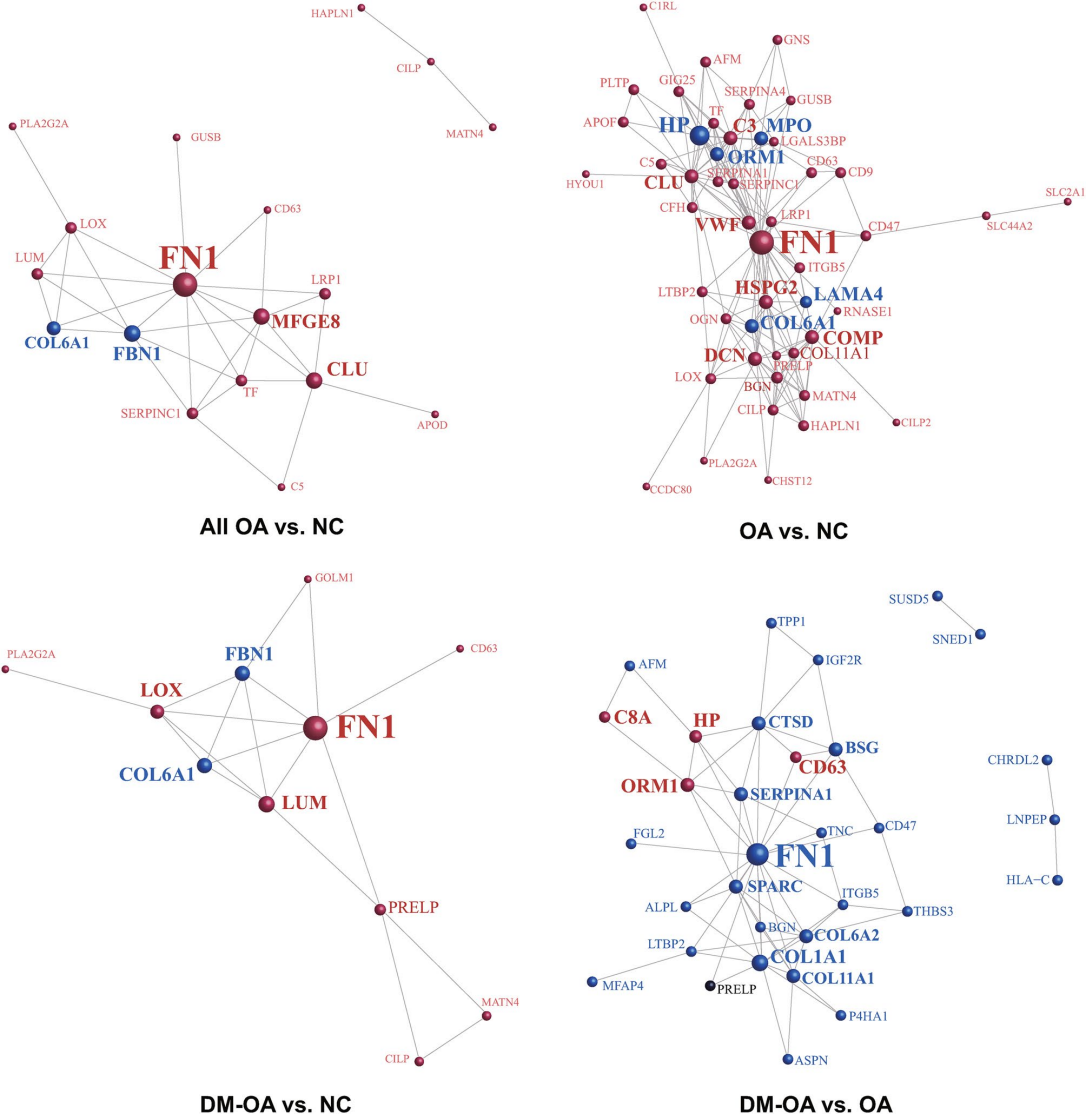
Protein–protein interaction (PPI) analysis of N-glycoprotein. The protein interaction network diagram drawn by Cytoscape software (http://www.cytoscape.org/, version 3.2.1) is based on the N-glycoproteins corresponding to the differentially expressed N-glycosylated peptides fragments. The nodes indicated the differentially expressed N-glycoprotein and the connecting lines indicated the interactions between proteins. Down-regulated proteins are shown in blue while upregulated genes are shown in red. Proteins possessing multiple N-glycopeptides which were oppositely regulated are marked in gray. The size of the circle indicates the number of proteins directly interacting with a protein.

Based on the STRING or InAct databases, Cytoscape software was used to construct a protein interaction network diagram for the N-glycoprotein corresponding to the N-glycosylated peptides with differential abundances identified in each comparison. In the PPI interaction network, high connectivity with other proteins usually plays a central role in the pathogenesis, which causes intra-group differences. As shown in Fig. 4, the most connected protein in all three comparisons was FN1, which was upregulated in the OA and DM-OA groups compared to the NC group, and was higher in the OA group than in the DM-OA group. In addition to FN1, clusterin and haptoglobin were also members of the top three connected proteins in the comparison of the OA and NC groups, which were replaced by protein-lysine 6-oxidase (LOX) and LUM in the DM-OA and NC comparisons. In the comparison of the OA and DM-OA groups, FN1, collagen type I alpha 1 chain (COL1A1), and collagen type VI alpha 1 chain (COL6A2) played the most central role in the affected PPI network.

## Discussion

This exploratory study comparatively analyzed the N-glycosylated proteomics of articular cartilage in subpopulations of OA, OA with T2DM, and trauma without either OA or DM. First, our results showed that compared with the articular cartilage of trauma patients, there were 10 upregulated and 2 downregulated N-glycosylated proteins in the articular cartilage of ALL-OA (DM-OA and OA) patients (Table 1). Among the 10 upregulated N-glycosylated proteins, cartilage intermediate layer protein 1 (CILP), immunoglobulin heavy constant mu (IGHM), and lumican (LUM) were the top three enriched proteins, while fibrillin-1 (FBN1) and collagen type VI alpha 1 chain (COL6A1) were the two downregulated proteins. Most of the 12 proteins have previously been related to OA, including CILP^22^, LUM24, lactadherin (MFGE8)^24^, FBN1^22,25^, COL6A1^26^, apolipoprotein d (APOD)^27^, fibronectin 1 (FN1)^28^, and clusterin (CLU)^29^. Although the small sample size reduced the power of this study, the high agreement between our results and other reports supported the reliability of the method used. Moreover, our results imply that these proteins probably participate in the pathogenesis of OA in an N-glycosylated form.

In addition, the alteration of N-glycosylated proteins shared by patients with OA with and without DM might suggest the core pathogenesis of OA. We showed that upregulation of N-glycosylated FN1 and CILP, as well as downregulation of COL6A1, could be the principal N-glycosylation alterations in both OA and DM-OA subsets. FN1 is an important component of the ECM. Many studies have reported that the occurrence of OA is closely related to changes in FN1 mRNA^30,31^. The PPI network also confirmed that FN1 is a key gene closely related to the growth of synovial fibroblasts, which affects the onset of OA^32^. CILP was one of the few upregulated cartilage matrix proteins in the early and late stages of OA^33–35^. Genetic analysis revealed that a single nucleotide polymorphism (SNP) in CILP is closely related to OA^36^. COL6A1 has also been identified as a core gene in OA^26^. Those genes might be the core factors contributing to the pathogenesis of OA. In contrast, those genes identified with differential abundance exclusively in OA group, but not in DM-OA group, might probably be less involved in the pathogenesis of OA. As examples, there was no association between the incidence of OA and angiopoietin-related protein 2 (ANGPLT2), or cd63 antigen (CD63), which had been identified previously. ANGPTL2, a gene that was thought involved in the repair and remodeling of damaged tissues, has been recently shown to induce synovial inflammation^37^. Similarly, CD63 expresses at abnormally high levels on the surface of activated platelets and endothelial cells in inflamed tissue^38^. Both of them might be upregulated as a consequence of inflammation, but not indispensable initiators, in the pathogenesis of OA.

A comparison of N-glycosylated proteomics between T2DM-OA and OA subsets provided a new perspective for understanding the relationship between T2DM and OA. Surprisingly, it seemed that in T2DM patients, the upregulated expression of N-glycosylated FN1 in the OA group was downregulated in the DM-OA group, as well as some other N-glycosylated proteins. Interestingly, KEGG pathway analysis showed that the PI3K/Akt pathway (involving FN1, COL1A1, etc.) was downregulated in the DM-OA group compared to the OA group. As PI3K/Akt inhibition has been suggested as a treatment for OA^39,40^, whether the decrease in N-glycosylated proteins in the PI3K/Akt pathway inhibits the activity of the PI3K/Akt pathway and thereby exerts a protective effect against OA needs to be further investigated.

Most importantly, compared with the OA group, the level of the N-glycosylation complement component C8 alpha chain (C8α) N437 was significantly increased in the DM-OA group. Numerous studies have shown the role of complement activation in the pathogenesis of metabolic diseases including obesity, insulin resistance and diabetes^41^. As glycosylation of C8α N437 is involved in complement activation and the formation of complement complexes^41^, it is reasonable to deduce that the abnormally activated complement C8α in DM patients may also contribute to joint damage in OA. This hypothesis could explain the association between DM and the severity of OA, and further suggests a therapeutic strategy for preventing complement activation which should be considered in the treatment of OA patients with DM.

We are also aware of some limitations to the current study. First, as it is impossible to obtain cartilage tissue from healthy people due to ethical requirements, the cartilage of patients with acute traumatic joint injury was used as a normal control in our study. However, in these samples, the N-glycosylation profile may be affected by injury-associated inflammation. The drawbacks in our "normal control" may have inhibited us from identifying N-glycosylated proteins with differential abundance in the inflammatory process. Nevertheless, the results of the comparison between OA and DM-OA, which is the main purpose of our study, would be unaffected. Moreover, we observed no significant influence of specific drugs on N-glycosylated proteomics by hierarchical clustering analysis as shown in Fig. S5. Although the small sample size reduced the power of the study, this exploratory study showed a general view of N-glycosylated sites and proteins in the cartilage of OA patients with and without T2DM, provided new ideas for understanding the pathogenesis of OA, and suggested the possible contribution of T2DM to OA pathogenesis.

## Materials and methods

### Study design

The objective of this study was to characterize the human cartilage N-glycoproteome and to compare protein N-glycosylation aberrations and their affected biological processes in OA and OA complicated with T2DM (DM-OA). We used a mass spectrometry-based quantitative N-glycoproteomics pipeline^42,43^ to perform unbiased, large-scale, N-glycoproteome profiling analysis of human cartilage samples from OA and DM-OA patients, as well as from patients with acute traumatic joint injury, used as a normal control. N-glycopeptides containing ^18^O-tagged N-glycosylation sites were identified and quantified, and their glycopeptide abundances were normalized and subjected to differential abundance analysis to identify disease-related alterations in N-glycopeptides in OA and DM-OA.

### Participants and ethical approval

A total of 10 patients diagnosed with primary degenerative knee osteoarthritis with Kellgren-Lawrence grade 3 or 4^44^ who underwent unilateral knee arthroplasty at Zhongshan Hospital of Traditional Chinese Medicine, Guangdong Province, were enrolled in the study. In these patients, five cases were complicated with type 2 diabetes (DM-OA), while the others were not (OA group). All patients with DM-OA were treated with metformin and gliclazide before surgery to normalize their blood glucose levels. In addition, five patients with acute traumatic joint injury without DM or OA were enrolled as normal controls (NC). After obtaining informed consent, cartilage samples were taken, rinsed with saline, and flash frozen in liquid nitrogen immediately after surgery. This study was conducted in accordance with the principles of the Declaration of Helsinki and approved by the Ethics Committee of Zhongshan Hospital of Traditional Chinese Medicine, Guangdong Province (Approval Number 2020ZSZY-LLK-202).

### Protein extraction and digestion

An appropriate amount of cartilage sample was ground in liquid nitrogen. After TCA/acetone precipitation and washing, SDT buffer (4% SDS, 100 mM Tris/HCl pH7.6, 0.1 M DTT) was added, and the mixture was boiled for 15 min. After centrifugation at 14,000*g* for 40 min, the supernatant was quantified using the BCA Protein Assay Kit (Bio-Rad, USA). Proteins (200 μg) from each sample were incorporated into 30 μL SDT buffer (4% SDS, 100 mM DTT, 150 mM Tris–HCl pH 8.0). The detergent, DTT, and other low-molecular-weight components were removed using UA buffer (8 M Urea, 150 mM Tris–HCl pH 8.0) via repeated ultrafiltration (Microcon units, 10 kD). Then, 100 μL iodoacetamide (100 mM IAA in UA buffer) was added to block the reduced cysteine residues and the samples were incubated for 30 min in the dark. The filters were washed three times with 100 μL UA buffer and then 100 μL 25 mM NH_4_HCO_3_ buffer. Finally, the protein suspensions were digested with 4 μg trypsin (Promega, USA) in 40 μL 25 mM NH_4_HCO_3_ buffer overnight at 37 °C, and the resulting peptides were collected as a filtrate. The peptides from each sample were desalted on C18 cartridges (Empore SPE Cartridges C18 (standard density), bed I.D. 7 mm, volume 3 ml, Sigma, USA), concentrated by vacuum centrifugation and reconstituted in 40 µL of 0.1% (v/v) formic acid. The peptide content was estimated by UV light spectral density at 280 nm using an extinction coefficient of 1.1 of 0.1% (g/l) solution, which was calculated based on the frequency of tryptophan and tyrosine in vertebrate proteins.

### Lectin enrichment and deglycosylation in H2O^18^

The digested peptides were mixed with a lectin solution containing a combination of ConA, WGA, and RCA 120 (Sigma, USA) at ratio of 2:1 (protein to lectin, w/w). The mixtures were then transferred to new YM-30 filter units. After 1 h of incubation at room temperature, unbound peptides were eluted by centrifugation. To avoid false positives caused by deamidation, the captured peptides were washed with binding buffer and NH_4_HCO_3_ buffer in H_2_O^18^ three times before PNGaseF was added. Finally, the filter units were transferred to a new tube, 3 µg PNGaseF in 40 µl 25 mM NH_4_HCO_3_ in H_2_O^18^ was added to the filter units. After incubation, deglycosylated peptides were eluted by centrifugation.

### LC–MS/MS-based N-glycoproteomics analysis

LC–MS/MS analysis was performed on a Q Exactive HF/HFX mass spectrometer (Thermo Scientific) coupled with an Easy nLC (Proxeon Biosystems, now ThermoFisher Scientific) for 120 min. The peptides were loaded onto a reverse-phase trap column (Thermo Scientific Acclaim PepMap100, 100 μm * 2 cm, nanoViper C18) connected to a C18-reversed-phase analytical column (Thermo Scientific Easy Column, 10 cm long, 75 μm inner diameter, 3 μm resin) in buffer A (0.1% Formic acid) and separated with a linear gradient of buffer B (84% acetonitrile and 0.1% Formic acid) at a flow rate of 300 nl/min controlled by IntelliFlow technology. The mass spectrometer was operated in the positive ion mode. MS data were acquired using a data-dependent top10 method dynamically choosing the most abundant precursor ions from the survey scan (300–1800 *m*/*z*) for HCD fragmentation. The instrument was run using the peptide recognition mode.

The raw MS data for each sample were combined and searched against the human UniProt TrEMBL database using MaxQuant software (version number 1.5.3.17)^45^ for identification and quantitation analysis. The following parameters were used: 20-ppm precursor ion mass tolerance, trypsin digestion with up to two missed cleavages, fixed modification: cysteine carbamidomethylation (+ 57.0215 Da); variable modifications: asparagine deamidation in H2^18^O (18O tag of Asn, + 2.9890 Da), asparagine and glutamine deamidation (+ 0.9840 Da), methionine oxidation (+ 15.9949 Da), and N-terminal acetylation (+ 42.0106 Da). The false discovery rate (FDR) for peptide and protein identification was set at 1%. Only peptides with ^18^O-tagged asparagine residues within the N-glycosylation sequon N-X-S|T|C (X ≠ P) were accepted as true N-glycosite–containing peptides.

### Bioinformatic analysis

Cluster 3.0 (http://bonsai.hgc.jp/~mdehoon/software/cluster/software.htm) and Java Treeview software (http://jtreeview.sourceforge.net) were used to perform the hierarchical clustering analysis. The Euclidean distance algorithm for similarity measurement and average linkage clustering algorithm (clustering uses the centroids of the observations) for clustering were selected when performing hierarchical clustering. A heat map is often presented as a visual aid, in addition to the dendrogram. CELLO (http://cello.life.nctu.edu.tw/), a multiclass SVM classification system, was used to predict the subcellular protein localization. The protein sequences of the selected differentially expressed proteins were locally searched using the NCBI BLAST + client software (ncbi-blast-2.2.28 + -win32.exe) and InterProScan to identify homolog sequences, then gene ontology (GO) terms were mapped, and sequences were annotated using the software program Blast2GO. Following annotation, the studied proteins were blasted against the online Kyoto Encyclopedia of Genes and Genomes (KEGG) database (http://geneontology.org/) to retrieve their KEGG orthology identifications and were subsequently mapped to KEGG pathways. Enrichment analysis was performed based on Fisher’ exact test, considering all quantified proteins as a background dataset. The Benjamini- Hochberg correction for multiple testing was further applied to adjust the derived *p*-values (< 0.05). The protein–protein interaction (PPI) information of the studied proteins was retrieved from the IntAct molecular interaction database (http://www.ebi.ac.uk/intact/) using their gene symbols or STRING software (http://string-db.org/). The results were downloaded in XGMML format and imported into Cytoscape software (http://www.cytoscape.org/, version 3.2.1) to visualize and further analyze the functional protein–protein interaction networks.

### Statistical analysis

A high-precision Q Exactive series mass spectrometer was used for qualitative analysis of each set of N-glycosylated label-free data during the collection process, which was performed with less than 1% FDR for peptides and proteins as the screening criterion. Differences in N-glycosylated peptide abundance between groups were determined using Wilcoxon non-parametric test. N-glycosylated peptides with different presence/absence statuses (presence in ≥ 3 samples in one group while absence in the other group) were also included for functional prediction analysis. Enrichment analyses were performed with a two-sided Fisher’s exact test, as indicated, to calculate the *P* values. The significance threshold was set as 0.05.

### Consent for publication

This manuscript has not been published or presented elsewhere in part or entirety and is not under consideration by another journal. We have read and understood your journal’s policies, and we believe that neither the manuscript nor the study violates any of these.

## Supplementary Information

Supplementary Information.

## Data Availability

The mass spectrometry proteomics data have been deposited to the ProteomeXchange Consortium (http://proteomecentral.proteomexchange.org) via the iProX partner repository with the dataset identifier PXD028753.
