# Supplementary material for: Protein N-glycosylation aberrations and glycoproteomic network alterations in osteoarthritis and osteoarthritis with type 2 diabetes
Source: Sci Rep. 2022 Apr 28;12:6977. doi: 10.1038/s41598-022-10996-1 (PMC9051103; doi:10.1038/s41598-022-10996-1)

Supplementary Materials for

Protein N-glycosylation aberrations and glycoproteomic network alterations in Osteoarthritis and Osteoarthritis with Type II diabetes

Journal: Scientific Reports

Yi Luo, Ziguang Wu, Song Chen, Huanhuan Luo, Xiaoying Mo, Yao Wang, Jianbang Tang*
*Corresponding author. Email: 85945915@qq.com

The PDF file includes:

Table S1 to S4
Figs. S1 to S5

SUPPLEMENTARY TABLES

Table S1. The clinical characteristics of participants on their admission.

Case	Gender	Age (yr)	Body weight(kg)	OA stage*	HbA1c (mmol/L)	FBG (mmol/L)	Medicines
**Osteoarthritis with type 2 diabetes (DM-OA)**							
1	Female	62	65	IV	11.4	15.63	Insulin, Metformin
2	Female	80	51.6	IV	6.9	6.5	Metformin,Gliclazide
3	Male	68	54.5	IV	8.3	7.7	Metformin,Gliclazide
4	Female	34	52.2	IV	7.6	12.9	Acarbose, Repaglinide, Insulin
5	Female	63	67	IV	7.3	7.8	Acarbose
Median (IQR)		63.0(62.0,68.0)	54.5(52.2,65.0)				
**Osteoarthritis (OA)**							
1	Female	70	64	IV	6.2	5.4	
2	Female	68	62	IV	5.9	4.6	
3	Male	66	74	III	6.2	4.57	
4	Male	62	87.5	IV	7.2	5.8	
5	Female	54	80	III	6.1	4.81	
Median (IQR)		66.0(62.0,68.0)	74.0(64.0,80.0)				
**Control (NC)**							
1	Female	41	-			5.96	
2	Female	67	53.4			5.6	
3	Male	34	67			5.25	
4	Male	30	58			5.41	
5	Male	55	62			5.08	
Median (IQR)		41.0(34.0,55.0)	60.0(56.9,63.3)				

HbA1c: glycosylated hemoglobin; FBG: fasting blood glucose. *: Kellgren-Lawrence grade.

Table S2. N-glycosylated peptides with differential abundance identified in each pairwised comparison.

All-OA/NC (*n*=15)			OA/NC (*n*=23)			DM-OA/NC (*n*=6)			DM-OA/OA (*n*=17)		
Protein Gene Name	Positions within Proteins	All-OA/NC	Protein Gene Name	Positions within Proteins	OA/NC	Protein Gene Name	Positions within Proteins	DM-OA/NC	Protein Gene Name	Positions within Proteins	DM-OA/OA
CILP	346	10.65453	CILP	346	14.27352	CILP	346	6.130803	C8A	437	2.338182
IGHM	46	7.662703	FN1	542	5.430545	IGHM	46	4.419722	CD47	73	0.469331
CILP	129	5.226345	CILP	129	4.721511	LUM	160	2.840258	ANGPTL2	164	0.461199
LUM	249	4.52859	RNASE1	104	4.418653	FN1	1007	2.733601	BSG	160	0.448893
FN1	542	4.391847	ARID4B	743	4.380733	FBN1	1581	0.286231	CTSD	263	0.440195
MFGE8	64	4.180061	HAPLN1	56	4.325497	COL6A1	804	0.216793	JCAD	662	0.43813
ARID4B	743	3.854487	IGA2	207;92	4.105416				FN1	528	0.425126
SERPINC1	224	3.805051	C3	85	4.061049				TNC	1018	0.419092
ARID4B	744	3.313284	ARID4B	744	4.019371				COL1A1	1365;79	0.410458
APOD	98	3.297239	CLU	103	3.644306				BGN	270	0.404599
CLU	86	3.055946	SERPINC1	224	3.566427				FBLN7	124	0.374379
FN1	1007	2.491803	FN1	528	3.478993				TNC	184	0.336147
CD63	150	2.280744	CLU	86	3.26583				THBS3	937	0.330238
FBN1	1581	0.407392	ANGPTL2	164	3.152998				IGHM	209;332	0.317383
COL6A1	804	0.23401	BGN	270	3.039588				ASPN	282	0.283583
			COMP	742	2.813893				COL6A2	785	0.22914
			HSPG2	1755	2.712298				SPARC	116	0.146252
			CD63	150	2.667694						
			CFH	1029	2.425409						
			IGG1	299;180	2.260056						
			FN1	1007	2.250004						
			CD47	73	2.201686						
			COL6A1	804	0.255532						

***: The criteria were change folds > 2 (for up-regulation) or < 0.5 (for down-regulations) and *P* < 0.05 (T-test or Wilcoxon non-parametric test).**

Table S3. The number of differentials expressed N-glycosylated peptides identified in each comparison.

Comparisons	Significantly changing in abundance*			Consistent presence (in ≥ 3 cases)/absence[#]			Over all
	Upregulated	Downregulated	All	Upregulated	Downregulated	All	
ALL OA vs. NC	13	2	15	13	0	13	28
OA vs. NC	22	1	23	45	5	50	73
DM-OA vs. NC	4	2	6	8	0	8	14
DM-OA vs. OA	1	16	17	5	23	28	45

Note: "Differentially expressed N-glycosylated peptides" were identified with criteria of either "significantly changing in abundance" or "consistent presence/absence" with criteria of: (*) change folds > 2 (for up-regulation) or < 0.5 (for down-regulation) and $P < 0.05$ (T-test or Wilcoxon non-parametric test) or (#) presence in 3 or more samples (more than half) in one group whereas absence in the other group.

Table S4. N-glycosylated proteins involved in the top 3 enriched KEGG pathways of each comparison.

Map_ID	Map_Name	Protein Number	Protein name
**All OA vs. NC**			
hsa04151	PI3K-Akt signaling pathway	3	FN1, COL6A1, PDGFC
hsa04510	Focal adhesion	3	FN1, COL6A1, PDGFC
hsa04610	Complement and coagulation cascades	3	SERPINC1, C5, CLU
**OA vs.NC**			
hsa04512	ECM-receptor interaction	8	FN1, COL6A1, vWF, ITGB5, COMP, LAMA4, HSPG2, CD47
hsa04151	PI3K-Akt signaling pathway	7	FN1, COL6A1, vWF, ITGB5, COMP, LAMA4, PDGFC
hsa04510	Focal adhesion	7	FN1, COL6A1, vWF, ITGB5, COMP, LAMA4, PDGFC
**DM-OA vs. NC**			
hsa05205	Proteoglycans in cancer *	3	FN1, CD63, LUM
hsa04151	PI3K-Akt signaling pathway	2	FN1, COL6A1
hsa04510	Focal adhesion	2	FN1, COL6A1
**DM-OA vs.OA**			
hsa04512	ECM-receptor interaction	7	COL1A1, FN1, COL6A2, ITGB5, TNC, THBS3, CD47
hsa05165	Human papillomavirus infection	7	COL1A1, FN1, COL6A2, ITGB5, TNC, THBS3,HLA-C
hsa04151	PI3K-Akt signaling pathway	6	COL1A1, FN1, COL6A2, ITGB5, TNC, THBS3

Abbreviations:

C5: complement C5; CLU: clusterin; COL1A1: collagen type I alpha 1 chain; COL6A1: collagen type VI alpha 1 chain; COL6A2: collagen type VI alpha 2 chain; COMP: cartilage oligomeric matrix protein; FN1:fibronectin 1; HLA-C: major histocompatibility complex, class I, C; HSPG2: heparan sulfate proteoglycan 2; ITGB5: integrin subunit beta 5; LAMA4: laminin subunit alpha 4; LUM: lumican; PDGFC: platelet derived growth factor C; SERPINC1: serpin family C member 1; THBS3: thrombospondin 3; TNC: tenascin C; vWF: von Willebrand factor; PLA2G2A: phospholipase A2 group IIA. * $P<0.05$ by Fisher exact test.

SUPPLEMENTARY FIGURES

Fig.S1. Protein LRP1 (Prolow-density lipoprotein receptor-related protein 1) contains up to 21 N-glycosylation sites.

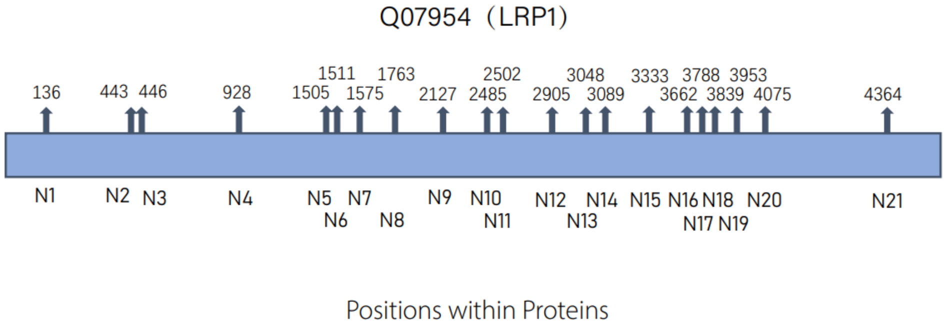

***N: N-glycosylation sites.**

Fig.S2. The Venn diagram of five individuals in each group.

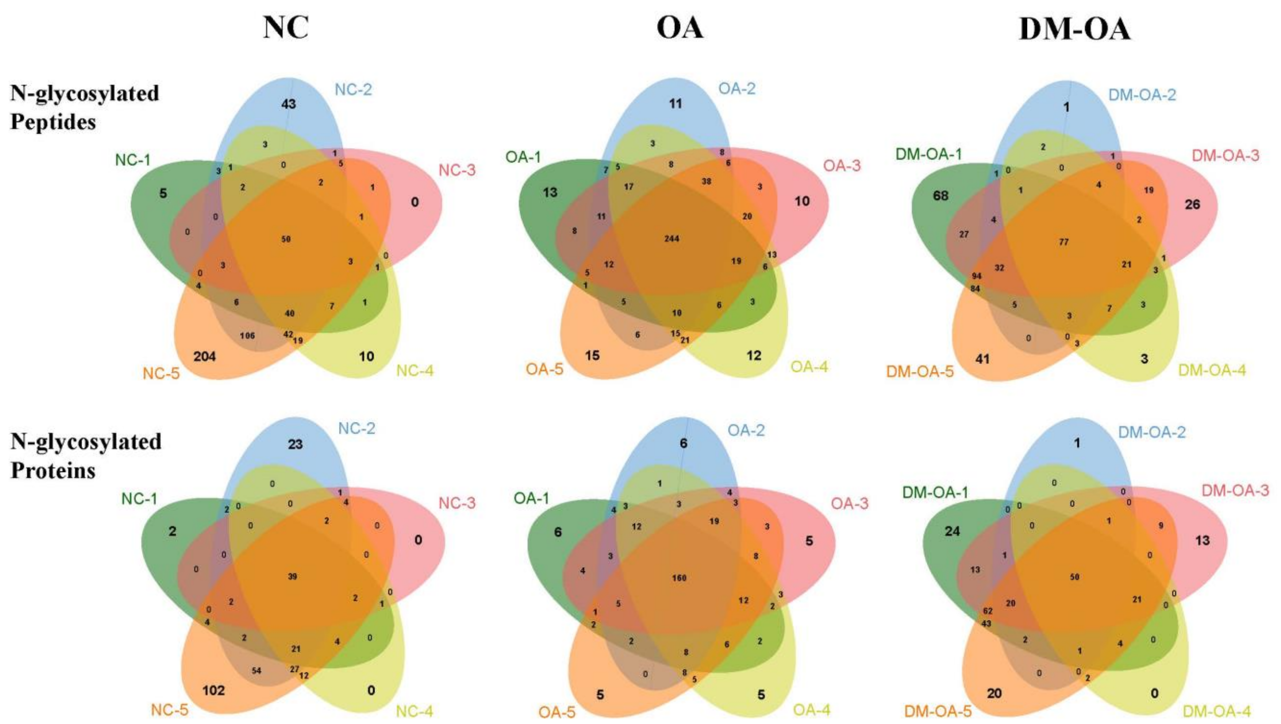

Fig.S3.GO analysis of N-glycosylated proteins differential expressed in OA and DM-OA patients.

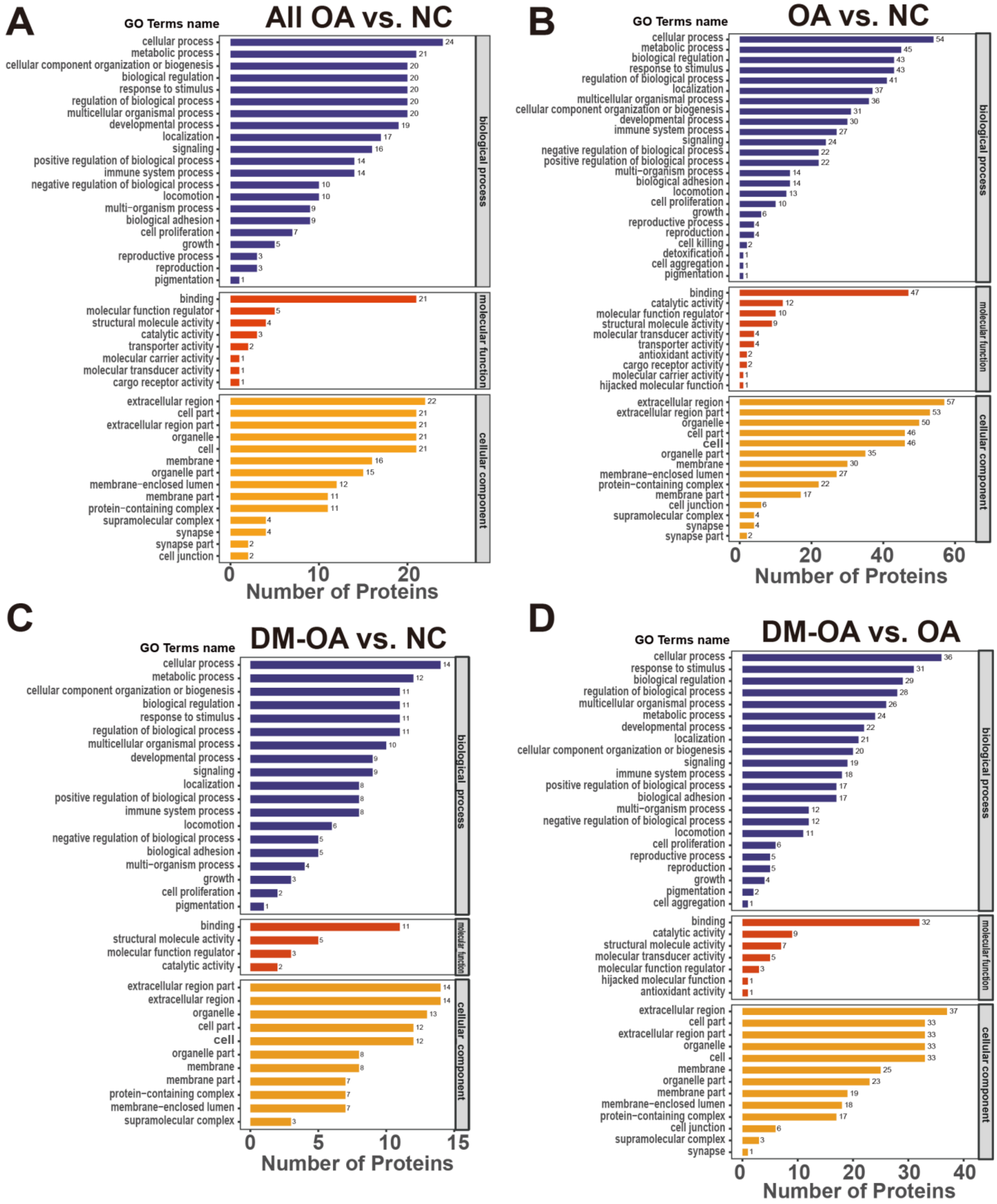

Fig.S4. Bubble chart of the top 20 enriched KEGG (Kyoto Encyclopedia of Genes and Genomes) pathways ranked by *P* values.

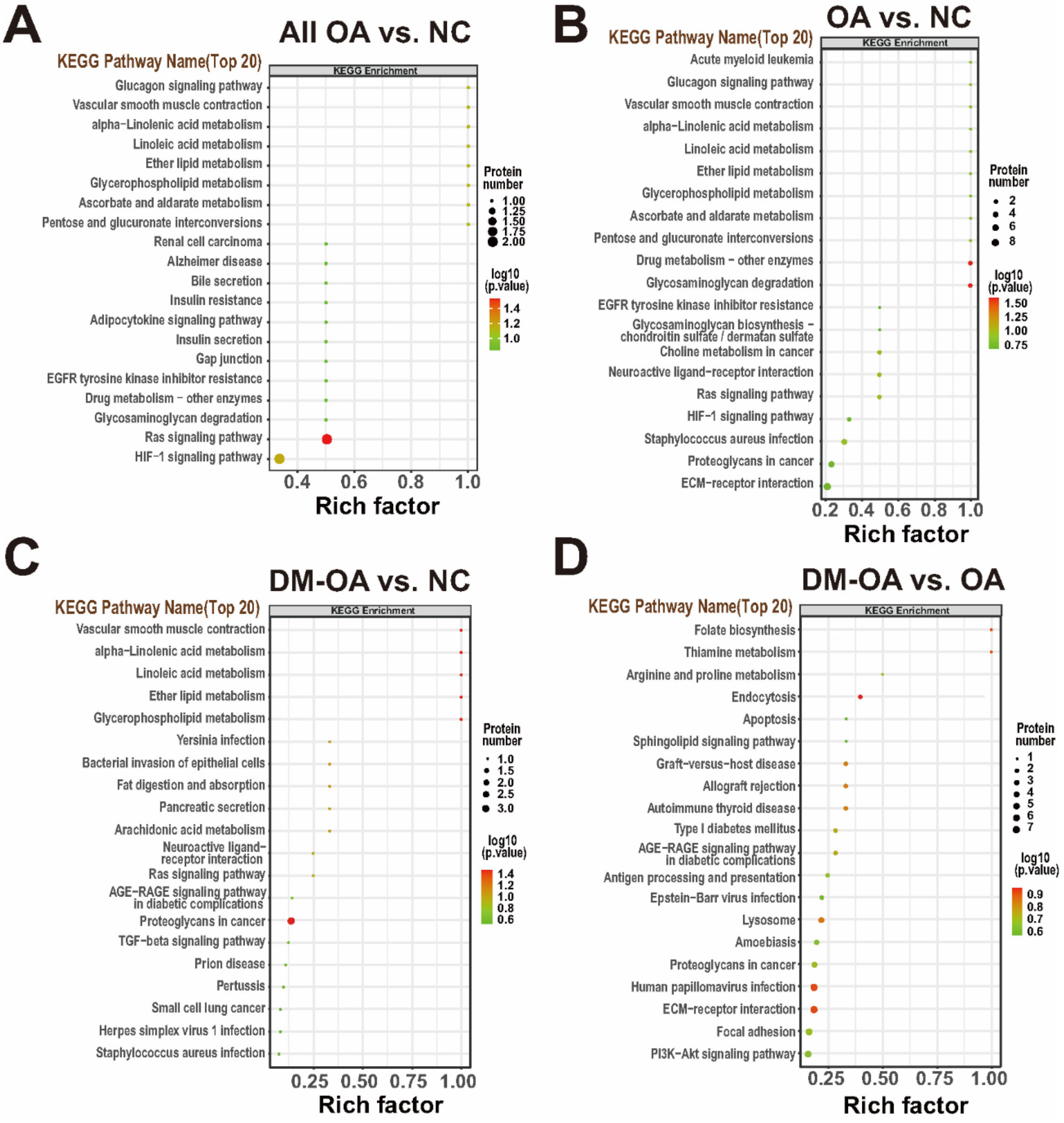

Note: The x-axis represents the enrichment factor (Rich Factor ≤ 1), and the bubble color indicated the logarithmic transformed P-value calculated by Fisher's Exact Test. With the criteria of $P<0.05$, Ras signaling pathway (consists of PLA2G2A and PDGFC) was enriched in "ALL OA vs. NC" comparison; Glycosaminoglycan degradation (consists of GUSB and GNS) and Drug metabolism (consists of GUSB and MPO) were enriched in "OA vs. NC" comparison; Proteoglycans in cancer (consists of FN1, CD63, and LUM) and five other KEGG pathways (all consist of only PLA2G2A) were enriched in "DM-OA vs. OA" comparison.

Abbreviations: GNS, glucosamine (N-acetyl)-6-sulfatase. GUSB, glucuronidase beta. MPO, myeloperoxidase.

Fig.S5. Hierarchical clustering of patients in OA group and DM-OA group showed irrelevance with DM medicines.

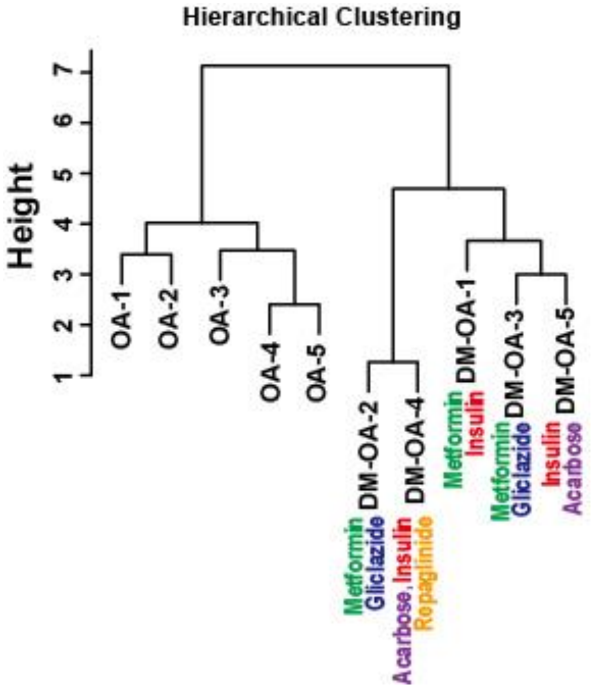

Supplement: Supplementary file 1 — Supplementary Information. [file 41598_2022_10996_MOESM1_ESM.pdf]
